# The *Vibrio cholerae* type VI secretion system employs diverse effector modules for intraspecific competition

**DOI:** 10.1038/ncomms4549

**Published:** 2014-04-01

**Authors:** Daniel Unterweger, Sarah T. Miyata, Verena Bachmann, Teresa M. Brooks, Travis Mullins, Benjamin Kostiuk, Daniele Provenzano, Stefan Pukatzki

**Affiliations:** 1 Department of Medical Microbiology and Immunology, University of Alberta, Edmonton, Canada AB T6G 2S2; 2 Department of Biological Sciences, University of Texas Brownsville, Brownsville, Texas 78520, USA; 3 Department of Biomedical Sciences, University of Texas Brownsville, Brownsville, Texas 78520, USA

## Abstract

*Vibrio cholerae* is a Gram-negative bacterial pathogen that consists of over 200 serogroups with differing pathogenic potential. Only strains that express the virulence factors cholera toxin (CT) and toxin-coregulated pilus (TCP) are capable of pandemic spread of cholera diarrhoea. Regardless, all *V. cholerae* strains sequenced to date harbour genes for the type VI secretion system (T6SS) that translocates effectors into neighbouring eukaryotic and prokaryotic cells. Here we report that the effectors encoded within these conserved gene clusters differ widely among *V. cholerae* strains, and that immunity proteins encoded immediately downstream from the effector genes protect their host from neighbouring bacteria producing corresponding effectors. As a consequence, strains with matching effector-immunity gene sets can coexist, while strains with different sets compete against each other. Thus, the *V. cholerae* T6SS contributes to the competitive behaviour of this species.

## Abstract (web summary)

Some strains of the pathogen *Vibrio cholerae* can kill each other by injecting effector proteins that are toxic in the absence of cognate ‘immunity’ proteins. Here, the authors show that strains with high pathogenic potential possess matching effector-immunity sets and can coexist.

## Graphical abstract

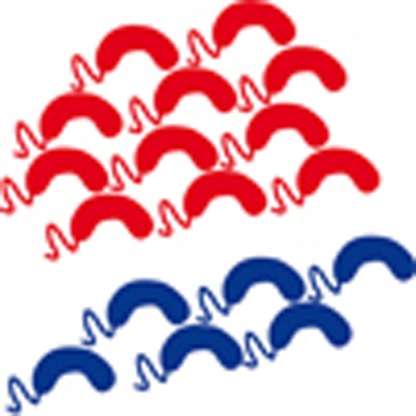

V*ibrio cholerae* is a Gram-negative bacterium predominantly found in marine environments^1^. *V. cholerae* comprises more than 200 highly diverse serogroups, but only toxigenic O1 serogroup strains that encode the virulence factors cholera toxin (CT) and toxin-coregulated pilus (TCP) have caused pandemic spread of the diarrhoeal disease cholera^2^,^3^,^4^. TCP biosynthesis genes are encoded within *Vibrio* pathogenicity island-1 (VPI-1), and TCP protruding from the bacterium serves as a receptor for a filamentous bacteriophage (CTX-ф) that transduces CT genes into the chromosomes of pandemic *V. cholerae* strains^5^. The contribution of CTX-ф is crucial to pathogenesis as only *V. cholerae* strains encoding CT are capable of pandemic spread^5^. When humans consume contaminated water, toxigenic *V. cholerae* passes through the gastric acid barrier, expresses TCP, secretes CT and colonizes the small intestine. Next, *V. cholerae* multiplies rapidly and exits the human host during diarrhoeal purges^6^. If left untreated, subsequent dehydration of the host can lead to death. During the presently ongoing seventh pandemic, approximately five million infections and 120,000 deaths due to cholera are estimated to occur each year^7^. Rehydration therapy is the preferred treatment, whereas the use of antibiotics is limited because of the emergence of antibiotic-resistant strains^8^.

In addition to the principal virulence factors TCP and CT, *V. cholerae* genomes encode the type VI secretion system (T6SS), a contact-dependent toxin delivery system^9^,^10^. Genes encoding the T6SS are present in ~25% of all sequenced Gram-negative bacteria, and are found in all sequenced *V. cholerae* strains^11^. The T6SS is a molecular puncturing device employed by bacteria to translocate effectors into eukaryotic and prokaryotic cells^10^,^12^. The hallmark of the secretion system is an inner tube composed of haemolysin-coregulated protein (Hcp) polymers^13^. Similar to a mechanism found in tailed bacteriophages, contraction of an outer sheath around the inner Hcp tube causes the inner tube to be ejected^14^,^15^. Three *V. cholerae* T6SS effectors (the lipase TseL^16^,^17^, the membrane-disrupting VasX^18^ and the peptidoglycan-degrading VgrG-3 (refs 16, 19)) believed to be situated at the distal end of the ejected Hcp tube target prokaryotic cells. On T6SS-mediated effector transfer from one bacterium into another, TseL, VasX and VgrG-3 are inactivated in target cells that express corresponding immunity genes *tsiV1*, *tsiV2* and *tsiV3*, respectively. The immunity genes are encoded directly downstream of their corresponding effector gene as part of an effector-immunity pair in each of the three T6SS gene clusters, the large and the two small auxiliary gene clusters (Fig. 1 and refs 16, 19).

We first noticed that *V. cholerae* strains compete against each other in a T6SS-dependent manner when we discovered that *V. cholerae* strains from the Rio Grande river delta (Southwestern United States) are able to kill each other^20^; however, the molecular mechanism and the biological significance of this intraspecies competition remained unclear. Here we apply a bioinformatics approach to identify the molecular basis of T6SS-mediated (in)compatibility. This analysis allows us to subdivide this bacterial species into strains that are able to coexist (compatible strains) and strains that outcompete each other in a T6SS-dependent manner (incompatible strains). Phylogenetic analysis reveals that toxigenic (as defined by the presence of VPI-1 and CTX-Φ) *V. cholerae* strains cluster within a single group of compatible strains.

## Results

### Conserved T6SS clusters harbour diverse effector modules

**Figure 1:**
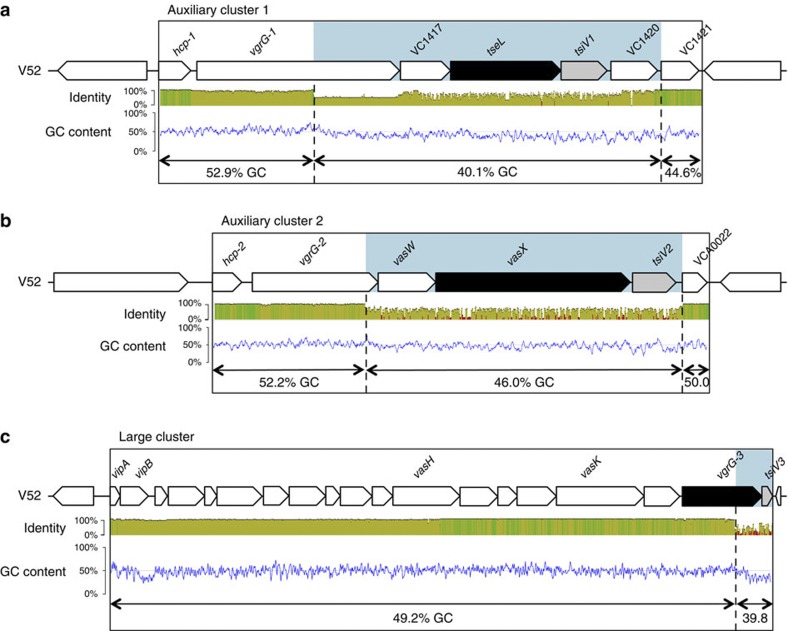
T6SS effector modules are highly diverse and differ in GC content. The boxes highlight auxiliary cluster 1 (**a**), auxiliary cluster 2 (**b**) and the large gene cluster (**c**) that encode the T6SS of *V. cholerae* V52. The results of a sliding-window analysis (with a window size of 10 nucleotides) in which the T6SS clusters of 37 *V. cholerae* strains were aligned and compared is indicated as average percent of nucleotide identity: 100% identity (green bars), at least 30% identity (yellow bars) and less than 30% identity (red bars). The GC content of *V. cholerae* V52 T6SS-encoding genes is plotted as the result of a sliding-window analysis (with a window size of 40). Average percentage of GC content was calculated for indicated regions. The T6SS effector genes for bacteria–bacteria interactions are shown in black and their cognate immunity genes are shown in grey. Regions of high diversity and low GC content are highlighted in blue.

We hypothesized that a given strain of *V. cholerae* deploys effectors to kill strains lacking cognate immunity proteins. Therefore, compatible strains do not kill each other because they express the same immunity proteins, whereas incompatible strains are unable to coexist because they kill each other as a result of having different effector and immunity gene pairs^21^. To test this hypothesis, the nucleotide sequences of the three T6SS clusters of 37 *V. cholerae* strains collected from a variety of sources over the last 77 years (Supplementary Table 1) were aligned and compared. As shown in Fig. 1, auxiliary clusters 1, 2 and the large cluster were found to be highly conserved among the available *V. cholerae* genomes except for regions of high diversity and low GC content (shaded blue) that encode the bacterial effectors TseL, VasX and VgrG-3, and their immunity proteins TsiV1, TsiV2 and TsiV3, respectively. Conserved T6SS genes contained an average GC content of ~51%, whereas the GC content in the region coding the effector-immunity pairs was an average of ~9% lower (Fig. 1), suggesting that these have a different origin than the remainder of the T6SS gene clusters and may have been acquired independently. We named these regions ‘effector modules’, each encoding a T6SS effector and its cognate immunity protein. The combination of all three effector modules in one strain comprises an effector module set.

### T6SS effector modules determine compatibility

**Figure 2:**
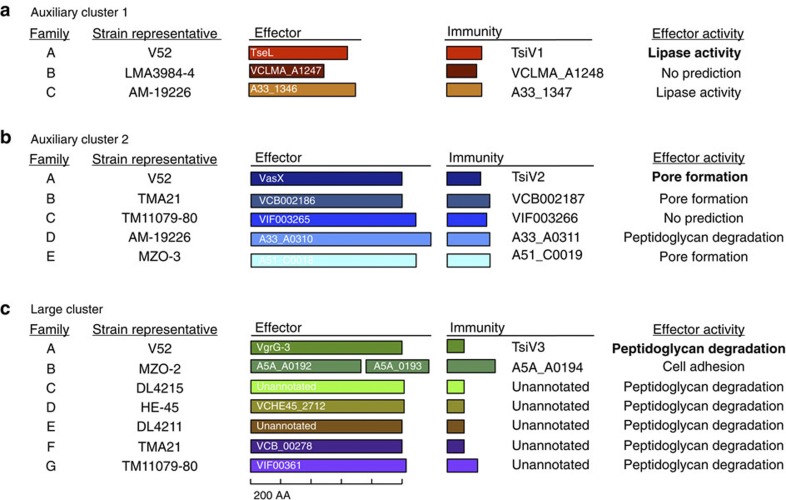
Families of *V. cholerae* T6SS effector modules. Effector and immunity proteins of individual families encoded in auxiliary cluster 1 (**a**), auxiliary cluster 2 (**b**) and the large cluster (**c**) are indicated by coloured boxes (box length is proportional to predicted protein length). The family of the effector module encoding the effector and immunity genes and a representative strain are shown on the left. Confirmed (bold type) or predicted (normal type) effector activities are shown on the right.

Effector modules at each of the three loci in the *V. cholerae* chromosomes were grouped into 15 families based on the amino-acid sequence identity of their immunity proteins TsiV1, TsiV2 and TsiV3; immunity proteins with >30% identity were grouped as a family because proteins sharing more than 30% identity are predicted to fold similarly^21^. As shown in Fig. 2, distinct families of effector modules at each locus were labelled alphabetically: three families were found in auxiliary cluster 1 (A–C), five in auxiliary cluster 2 (A–E) and seven in the large cluster (A–G), allowing the hypothetical existence of 105 different effector module sets. This is a conservative estimate, because our analysis does not account for the existence of additional, yet unknown effector modules.

**Figure 3:**
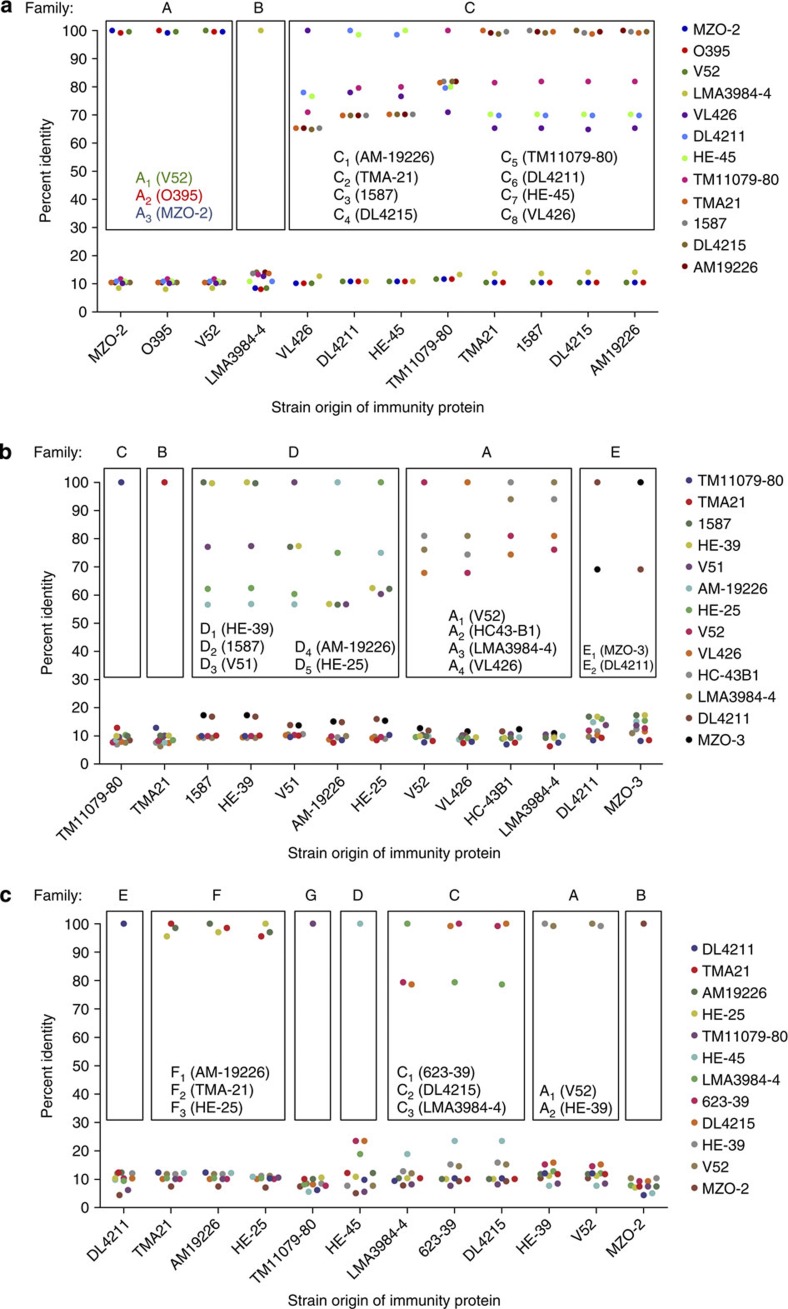
Pairwise comparison of TsiV1, TsiV2 and TsiV3 immunity proteins encoded in the T6SS effector modules. The names of the strains that harbour the analysed immunity proteins in their auxiliary cluster 1 (**a**), auxiliary cluster 2 (**b**) or the large cluster (**c**) are shown on the *x* axis and in the legend on the right. Percent sequence identity between two pairwise-aligned amino-acid sequences is indicated on the *y* axis. Immunity proteins with more than 30% sequence identity were grouped into families (indicated by boxes). Subfamilies are indicated within the boxes: each dot indicates a single subfamily defined by an identical amino-acid sequence. One representative is shown for each subfamily.

Effector module families also comprise multiple subfamilies, each consisting of identical immunity proteins and effectors that can display varying degrees of polymorphisms. Subfamilies are indicated by a subscript (for example, A_2_) as shown in Fig. 3 and Supplementary Fig. 1. Effector module sets of *V. cholerae* strains were labelled with a three-letter code (for example, A_1_A_1_A_1_) representing distinct families/subfamilies in T6SS auxiliary cluster 1, auxiliary cluster 2 and large cluster, respectively. Thus, we hypothesized that immunity proteins form the basis of (in)compatibility, allowing strains with polymorphic effectors to be compatible as long as these effectors are paired with identical immunity proteins.

**Figure 4:**
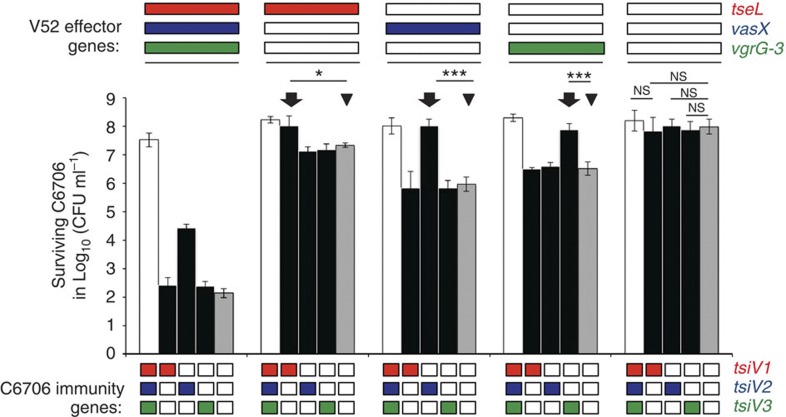
Cognate pairs of effector and immunity proteins act independently of each other. A competition assay of V52 effector mutants (top) against immunity mutants of C6706 (bottom) at a 10:1 ratio was performed. The logarithm of the number of surviving wild-type or mutant C6706 colony-forming unit is shown on the *y* axis. White, black and grey bars represent C6706 containing all three, one or no immunity protein-encoding genes, respectively. In-frame gene deletions are indicated by an empty rectangle. Triangles indicate toxic activity of individual T6SS effectors. Arrows highlight protection from the effector by its cognate immunity gene. Log-transformed data of two independent experiments each performed in duplicate are the basis for the arithmetic mean and the s.d. shown as bars and error bars, respectively. Stars indicate statistical significance (unpaired, two-tailed Student’s *t*-test: *P<0.05; ***P<0.0005; NS, *P*>0.05).

To test whether the effector module sets determine compatibility between strains, we chose V52 and C6706 as A_1_A_1_A_1_ module set representatives. Under laboratory conditions, V52 expresses T6SS effector and immunity genes, whereas C6706 expresses only immunity genes^16^,^22^. Therefore, although C6706 is T6SS-effector silent, C6706 exhibits immunity towards V52. An immunity deletion mutant (Δ*tsiV1,* Δ*tsiV2* and Δ*tsiV3*) of T6SS-silent C6706 is viable under standard laboratory growth conditions, allowing us to study the genetic basis of compatibility between strains with the same effector module set. C6706 strains encoding one, none or all immunity genes (*tsiV1, tsiV2* and *tsiV3*) were challenged with V52 that express one, none or all T6SS effectors (*tseL*, *vasX* or *vgrG-3*). Each *V. cholerae* V52 mutant expressing any one of the three T6SS effectors killed a C6706 immunity mutant lacking all three immunity genes 10- to 100-fold (Fig. 4, triangles). In contrast, C6706 mutants encoding only one immunity gene survived in the presence of V52 equipped with only the single corresponding T6SS effector (Fig. 4, arrows). These results indicate that cognate effector and immunity gene pairs function independently of one another, and that all three immunity proteins are required for complete protection. *V. cholerae* strains that encode the same effector module sets are able to coexist and are compatible. Strains that lack cognate immunity genes are outcompeted and are thus incompatible.

### Strains with different effector module sets are incompatible

**Figure 5:**
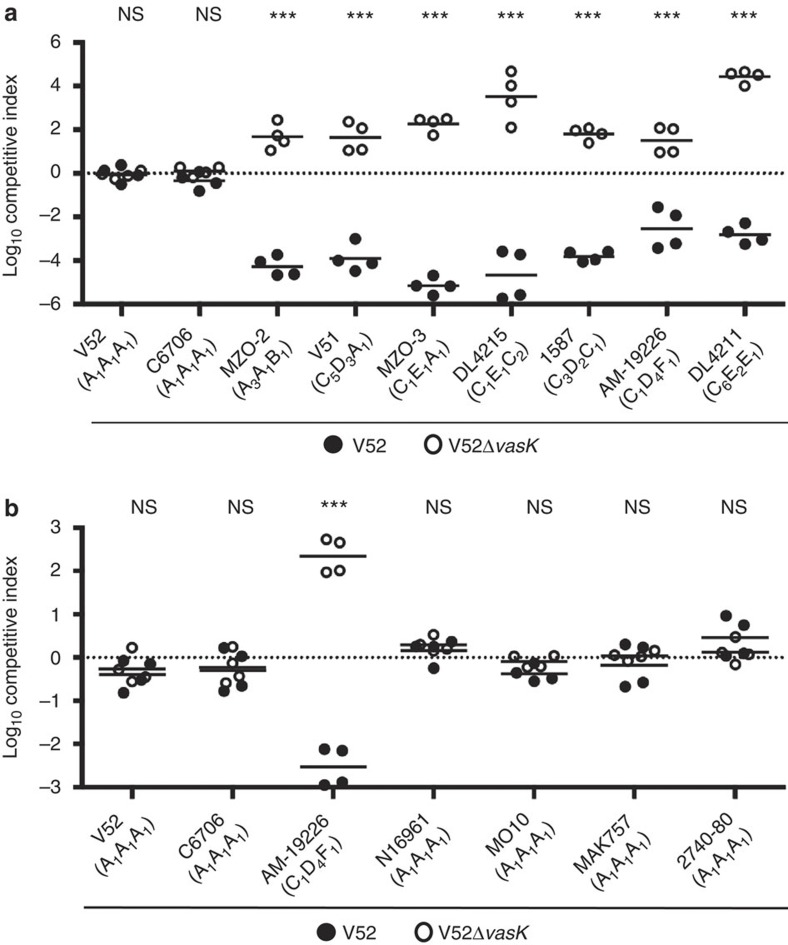
T6SS effector modules govern compatibility. T6SS diversity determines competition (**a**) and coexistence (**b**). Wild-type or *vasK*-deficient V52 was mixed with indicated *V. cholerae* strains; the mixture was incubated on nutrient agar for 4 h, and survival was enumerated by plating survivors on appropriate selective plates. The results of two independent experiments performed in duplicate are shown. Horizontal bars indicate the arithmetic mean of log-transformed data. Stars indicate statistical significance (unpaired, two-tailed Student’s *t*-test: ***P<0.0005; NS, *P*>0.05).

To challenge our compatibility model, we coincubated V52 (A_1_A_1_A_1_ effector module set) with T6SS-active strains (Supplementary Figs 2A and 3) harbouring the following effector module sets: A_1_A_1_A_1_ (C6706), A_3_A_1_B_1_ (MZO-2), C_5_D_3_A_1_ (V51), C_1_E_1_A_1_ (MZO-3), C_4_E_1_C_2_ (DL4215), C_3_D_2_C_1_ (1587), C_1_D_4_F_1_ (AM-19226) or C_6_E_2_E_1_ (DL4211) (Fig. 5a). In agreement with our hypothesis, all A_1_A_1_A_1_ strains tested were able to coexist (Fig. 5b), whereas non-A_1_A_1_A_1_ *V. cholerae* were effectively outcompeted by wild-type V52 when inoculated at a 1:1 ratio (Fig. 5a). Control experiments carried out with a V52 *vasK* in-frame deletion mutant unable to translocate effectors confirmed that killing was T6SS dependent (Fig. 5a,b).

**Figure 6:**
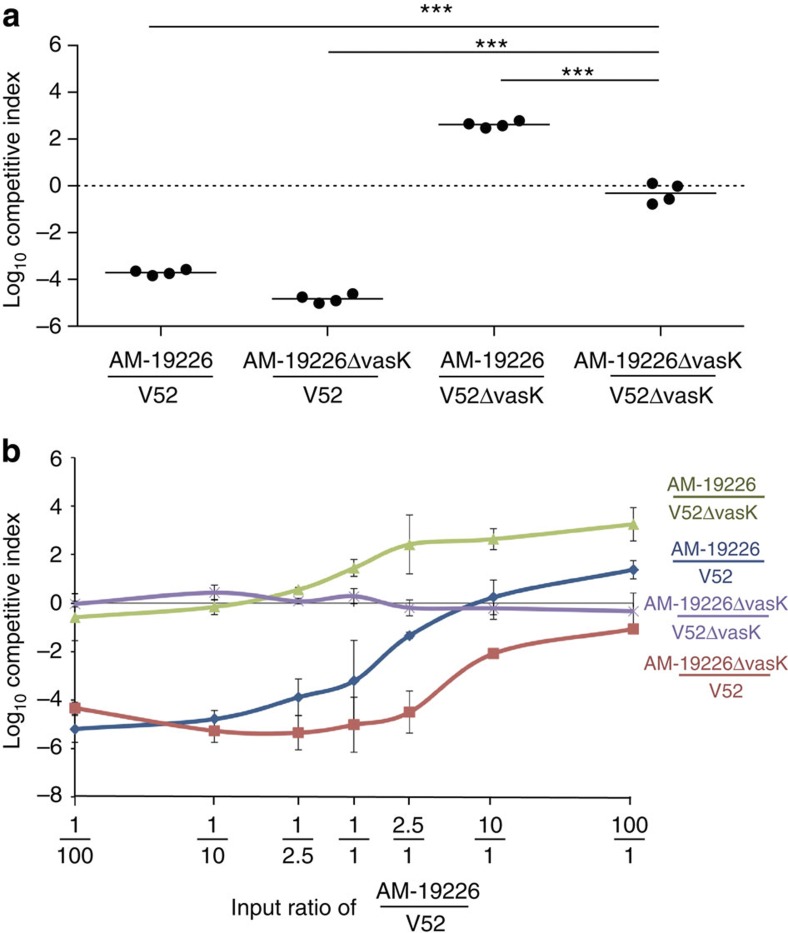
Incompatible strains engage in T6SS-mediated attacks. Wild-type or *vasK*-deficient V52 was mixed with wild-type or *vasK*-deficient AM-19226 at a ratio of 1:1 (**a**) or at various ratios as shown on the *x* axis (**b**). The logarithm of the competitive index of AM-19226 over V52 after 4 h coincubation is shown on the *y* axis. Horizontal bars indicate the arithmetic mean of log-transformed data based on two independent experiments, each performed in duplicate. Stars indicate statistical significance (unpaired, two-tailed Student’s *t*-test: ***P<0.0005). Error bars indicate the s.d. of log-transformed data.

To further examine the competitive interactions between A_1_A_1_A_1_ *V. cholerae* and strains that harbour a different effector module set, V52 (A_1_A_1_A_1_) and AM-19226 (C_1_D_4_F_1_) were inoculated at different ratios. Both strains exhibit constitutive T6SS activity under laboratory conditions as shown by secretion of the inner tube component Hcp and T6SS-dependent killing of *Escherichia coli* (Supplementary Figs 2B,C and 4). The two strains were coincubated at equal numbers on agar plates, and enumeration of survivors after 4 h showed that wild-type V52 outcompeted wild-type AM-19226 in a T6SS-dependent manner (Fig. 6a). When mixed at different input ratios, V52 remained competitive even when inoculated in the minority (Fig. 6b). In conclusion, *V. cholerae* strains with different effector module sets compete with each other and are thus incompatible.

### Toxigenic strains carry compatible T6SS effector module sets

**Figure 7:**
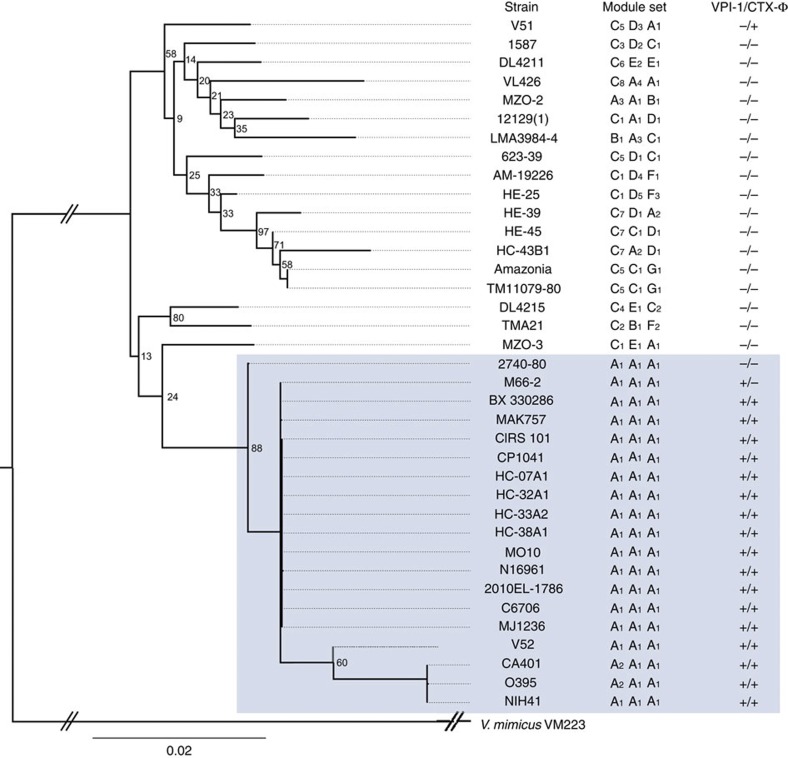
Phylogenetic relationship of *V. cholerae* strains with a variety of T6SS effector module sets. A neighbour-joining phylogenetic tree (bootstrap=100) of the 37 *V. cholerae* strains analysed based on the six housekeeping genes *adk, gyrB, mdh, recA, pgi* and *rpoB* is shown. Effector module sets indicating the family of the effector module in auxiliary cluster 1, auxiliary cluster 2 and the large cluster, and VPI-1/CTX-ф acquisition are indicated next to the strain name. Strains that harbour the AAA effector module set are highlighted in the blue box.

To determine the genetic relationship between compatible and incompatible strains, a phylogenetic tree for the 37 *V. cholerae* strains (Supplementary Table 1) used in this study was built based on six polymorphic housekeeping genes^23^ (Fig. 7). TCP was found exclusively in AAA strains. CTX-Φ was present in all but two AAA strains (2740-80, M66-2, both belonging to the O1 serogroup) and in one non-AAA strain, V51. Except V51—a clinical O141 serotype strain from the United States, all non-AAA strains analysed were VPI-1- and CTX-Φ-negative. Toxigenic strains (including C6706, MO10, V52, N16961 and O395) have the AAA effector module set, while *V. cholerae* strains with any other effector module set belong to phylogenetic lineages that exclude toxigenic strains.

## Discussion

This report describes a diverse set of genetic elements within the T6SS gene clusters of *V. cholerae* strains that we named effector modules. These modules differ in their GC content from the remainder of the clusters and encode T6SS effector and immunity genes. The three effector modules comprise an effector module set characteristic for each *V. cholerae* strain. Our data suggest that *V. cholerae* strains with the same effector module set can coexist and are therefore compatible, whereas when strains with different effector module sets engage their T6SS, one or both will experience cell death resulting in incompatibility. Compatibility between strains based on effector modules of the same family needs to be further explored as immunity proteins can differ within a family. We speculate that compatibility between strains decreases as the diversity between their immunity proteins increases. Conversely, incompatible strains might coexist under conditions that do not support production of all T6SS effectors, or as a result of cross-immunity^24^ provided by homologous immunity proteins.

Our study identified a wide variety of effector modules encoded within *V. cholerae* T6SS gene clusters. The differences in GC content between genes of the effector modules and genes that encode structural T6SS components suggest independent acquisition and integration events into the genome. This hypothesis is also supported by phylogenetic evidence (Fig. 7). For example, despite having undergone 162 genetic changes in the housekeeping genes since diverging from their last common ancestor (Fig. 7, Supplementary Fig. 5A), strains V51 and VL426 carry an identical copy of the immunity gene *tsiV3*^*A1*^ (the family of the effector module that encodes *tsiV3* is indicated in superscript; Supplementary Fig. 5B); sequence identity indicates that V51 and VL426 recently acquired the *tsiV3*^*A1*^ allele horizontally. Similarly, strains MZO-2 and 12129(1) are distantly related to O1 strains (Fig. 7), but carry an identical *tsiV2*^*A1*^ allele (Supplementary Fig. 6), strongly supporting the notion that *tsiV2*^*A1*^ was recently acquired by horizontal gene transfer. Evidence for these and other immunity gene transfer events can be found for any of the three effector modules, suggesting that horizontal acquisition of effector modules has been a recurrent theme in *V. cholerae* evolution. Furthermore, a phylogenetic tree constructed from six housekeeping genes (Fig. 7) revealed that strains derived from the same recent common ancestors frequently carry distinct effector module sets, suggesting that horizontal movement of effector modules, at least in some cases, occurred sometime after differentiation into distinct strains. For example, DL4215 (C_4_E_1_C_2_) and TMA21 (C_2_B_1_F_2_) share a recent common ancestor but differ in two of the three effector modules. Analogously, AAA strains share a common ancestor with MZO-3 (C_1_E_1_A_1_), yet differ in two effector modules. In these lineages, the effector module sets of closely related strains are not a result of recent common ancestry, but probably a result of subsequent horizontal effector module acquisition. In these cases, the divergence of newly evolving strains from a common ancestor might have been accelerated by the acquisition of different effector modules. T6SS-mediated competition interferes with cell–cell contact and therefore possibly affects contact-dependent exchange of genetic elements. Subsequently, such events may be restricted to bacteria that encode the same effector module sets and represent one way for a newly evolving strain to separate from its ancestor.

The cluster of strains with the AAA effector module sets (blue-shaded portion of Fig. 7) consists of strains that differ in their VPI-1/CTX content (+/+, +/− and −/−) suggesting that toxigenic and by extension pandemic *V. cholerae* acquired VPI-1 (and in some cases CTX-Φ) after AAA effector modules. As shown in Fig. 7, this holds true regardless of classical (responsible for the sixth pandemic and probably for the first five pandemics^25^,^26^,^27^) or El Tor biotype (responsible for the current seventh pandemic^28^). This indicates that AAA acquisition also preceded biotype differentiation and that toxigenic strains encode the AAA effector module set as a result of common ancestry. The conservation of the AAA effector module set over the evolutionary distance separating classical and El Tor biotypes suggests that natural selection could have pressured toxigenic strains to retain the contributions of the AAA module set because it is advantageous to human colonization (as they are likely to come in close contact with *V. cholerae* employing other effectors in the small intestine). Other strains such as MZO-2 (A_3_A_1_B_1_) and 623-39 (C_5_D_1_C_1_) separated by similar evolutionary distances have experienced changes in all three effector modules (Fig. 7). These observations, including that none of the toxigenic strains included in this study have acquired non-A modules, suggest that natural selection favours the AAA effector module set in toxigenic strains. T6SS-mediated competition of *V. cholerae* occurs *in vivo*^29^; thus, intraspecies competition might allow AAA strains to outcompete incompatible *V. cholerae* strains on host entry to establish a colonization niche. In such a scenario, AAA strains similar to 2740-80 lacking TCP and CT might be viewed as cheaters^30^,^31^,^32^ because when gaining access to a host along with a toxigenic AAA strain, they could coexist and colonize successfully without producing virulence factors.

The discovery of diverse effector modules has translational implications because inhibition of T6SS immunity would not affect bacterial viability *per se*, and be detrimental only when the target bacterium comes in close contact with a T6SS-active competitor. Current antibiotic compounds target bacterial metabolism, cell wall and growth, applying selective pressure towards essential functions, thereby favouring the emergence of adaptive mechanisms^8^. As a result, mutations that give rise to antibiotic resistance give rise to the emergence of strains that complicate the treatment of bacterial infections. We suggest that antibacterial strategies against a contact-dependent killing mechanism conserved among toxigenic strains would apply less selective pressure than currently employed antibiotics that target essential cellular housekeeping functions. AAA immunity genes might represent particularly attractive targets because all toxigenic (TCP+/CT+) strains capable of causing pandemic cholera that we evaluated in our study carry the AAA effector module set. Finally, AAA effector module sets could be targeted as a biomarker for toxigenic strain identification for epidemic risk assessment. Classification of the *V. cholerae* strains based on effector module sets may affect future outbreak management, diagnostics and therapeutic approaches.

## Methods

### Bacterial strains and cell culture

*V. cholerae* was grown in Luria Bertani (LB) broth supplemented with either rifampicin, 50 μg ml^−1^; ampicillin, 100 μg ml^−1^; or streptomycin, 100 μg ml^−1^ as appropriate. An endonuclease-minus mutant of AM-19226 was used as a wild type^33^ and as the parent strain for the *vasK* deletion mutant. A complete list of strains used in this study and their sources is shown in Supplementary Table 2.

### Competition assay

This assay was performed as previously described^10^. In brief, predator and prey bacteria were mixed at a 1:1 ratio unless otherwise stated. A total number of 2 × 10^8^ bacteria were spotted on LB plates and incubated at 37 °C for 4 h. Bacteria were resuspended, diluted and plated on LB plates containing antibiotics that allowed selection of predator or prey. Each experiment was done twice in duplicates. Colony-forming units per ml of V52 were divided by the colony-forming units per ml of the competing strain at time points *t*=0 h and *t*=4 h; the V52/competing strain ratio at *t*=4 h was divided by the V52/competing strain ratio at *t*=0 h to calculate the competitive index.

### DNA manipulation

In-frame deletion of effector genes, immunity genes and *vasK* was performed using pWM91-based knockout constructs^34^. A list of primer sequences used is shown in Supplementary Table 3.

### Bioinformatic analysis

Analysed genome sequences not generated by in-house Illumina NGS sequencing were obtained from NCBI or The Broad Institute databases (Supplementary Table 4). The GC content of *V. cholerae* V52 was analysed using Geneious (version 6.1.5). The T6SS gene clusters of the 37 *V. cholerae* strains were compared using the MAUVE alignment tool^35^ and visualized using Geneious. Prediction of effector functions was based on Phyre2 homology searches^36^. To group the strains by effector modules (Fig. 2), the amino-acid sequences of the immunity proteins were aligned using MUSCLE^37^. Immunity proteins with a percentage amino-acid identity of at least 30% were grouped into the same family^21^. Amino-acid sequences of immunity proteins within a subfamily are identical. The phylogenetic tree shown in Fig. 7 was assembled based on the concatenated nucleotide sequences of *adk* (adenylate kinase, locus tag VC0986), *gyrB* (DNA gyrase subunit, locus tag VC0015), *mdh* (malate dehydrogenase, locus tag VC0432), *recA* (recombinase A, locus tag VC0543), *pgi* (glucose-6-phosphate isomerase, locus tag VC0374) and *rpoB* (DNA-dependent RNA polymerase, locus tag VC0328) to establish the relatedness of *V. cholerae* to the corresponding genes from *V. mimicus* as the outgroup^23^. The data set containing these homologues had 38 sequences and 11,046 positions. Alignments were done using ClustalW^38^. All positions were determined to be homologous and were included in the alignment. jModelTest2 (refs 39, 40) was used to find the best model of nucleotide evolution for the sequences, incorporating corrections for invariable sites as well as a four-category gamma correction for rate variation when appropriate. For the tree shown in Fig. 7, a substitution model that accounted for both invariant sites and gamma-distributed rate heterogeneity (GTR+I+G) was chosen.

PhyML v. 2.4.4 (ref. 40) was used for maximum likelihood analysis, and to generate ML bootstrap values based on 100 pseudoreplicates of each data set. The phylogenetic tree shown in Fig. 7 is the PhyML consensus topology.

To determine the phylogeny of the *tsiV*^A^ nucleotide sequences in Supplementary Fig. 6, the nucleotide sequences of the indicated genes were aligned using MUSCLE. RAxML^41^ (boostrap=100) was used for the phylogenetic analysis.

### Protein secretion profile

Western blot samples were prepared and analysed as described previously^20^. In brief, bacteria were grown to mid-logarithmic phase, centrifuged, and supernatants and pellets were subject to SDS–PAGE followed by immunodetection with polyclonal rabbit anti-Hcp antiserum^42^ (diluted 1:500) and monoclonal mouse anti-DnaK antibody (Stressgen, diluted 1:15,000). Goat anti-mouse horseradish peroxidase and goat anti-rabbit horseradish peroxidase (both Santa Cruz, diluted 1:3,000) were used as secondary antibodies.

### Statistical analysis

The two-tailed Student’s *t*-test was used to determine statistical significance between indicated groups of data points. Before performing the *t*-test, variances within two groups were tested for equality using the *F*-test. In case of significantly different variances, the logarithmic values of the data points were used for the *t*-test. Additional information to the individual statistical analysis performed in this study can be found in Supplementary Table 5.

## Author contributions

D.U., S.T.M., V.B., T.M.B., T.M., B.K. and S.P. performed experiments. D.U., D.P. and S.P. designed experiments, interpreted results and wrote the paper.

## Additional information

**Accession codes:** Nucleotide sequences of *V. cholerae* T6SS gene clusters have been deposited in the GenBank nucleotide database under accession codes KF228943, KF228947, KF228950, KC955251, KF228941, KF228945, KF228942, KF228946, KF228949, KF228944, KF228948 and KF228951.

**How to cite this article:** Unterweger, D. *et al.* The *Vibrio cholerae* type VI secretion system employs diverse effector modules for intraspecific competition. *Nat. Commun.* 5:3549 doi: 10.1038/ncomms4549 (2014).

## Supplementary Material

Supplementary Information Supplementary Figures 1-6, Supplementary Tables 1-5 and Supplementary References
