# Supplementary material for: The Vibrio cholerae type VI secretion system employs diverse effector modules for intraspecific competition
Source: Nat Commun. 2014 Apr 1;5:3549. doi: 10.1038/ncomms4549 (PMC3988814; doi:10.1038/ncomms4549)
Supplement: Supplementary Information — Supplementary Figures 1-6, Supplementary Tables 1-5 and Supplementary References [file ncomms4549-s1.pdf]

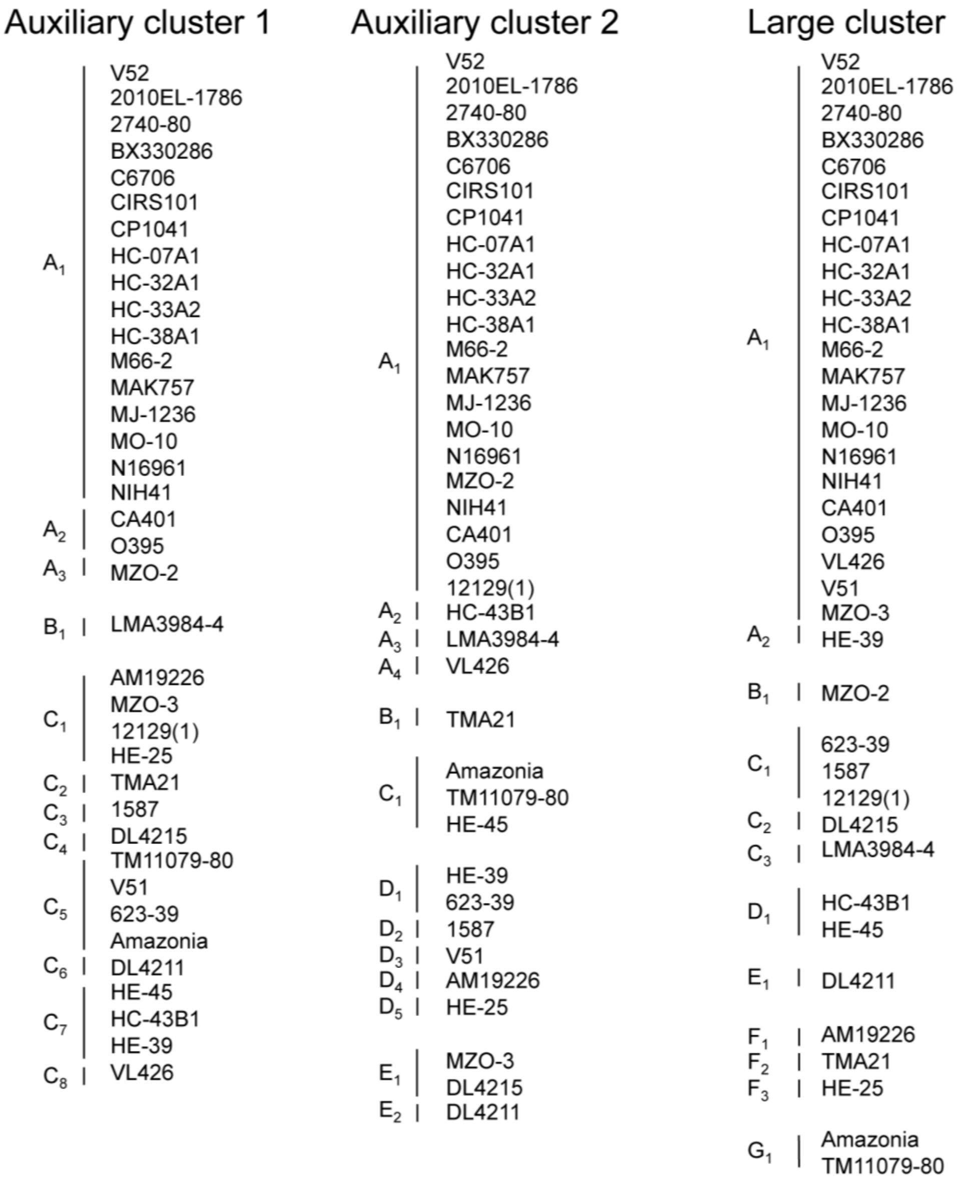

Supplementary Figure 1. Distribution of effector module families among the 37 analyzed *V. cholerae* strains. Families/subfamilies of the effector modules in auxiliary cluster 1 (A), auxiliary cluster 2 (B) and the large cluster (C) are shown on the left. Strains that encode the indicated effector modules are shown on the right.

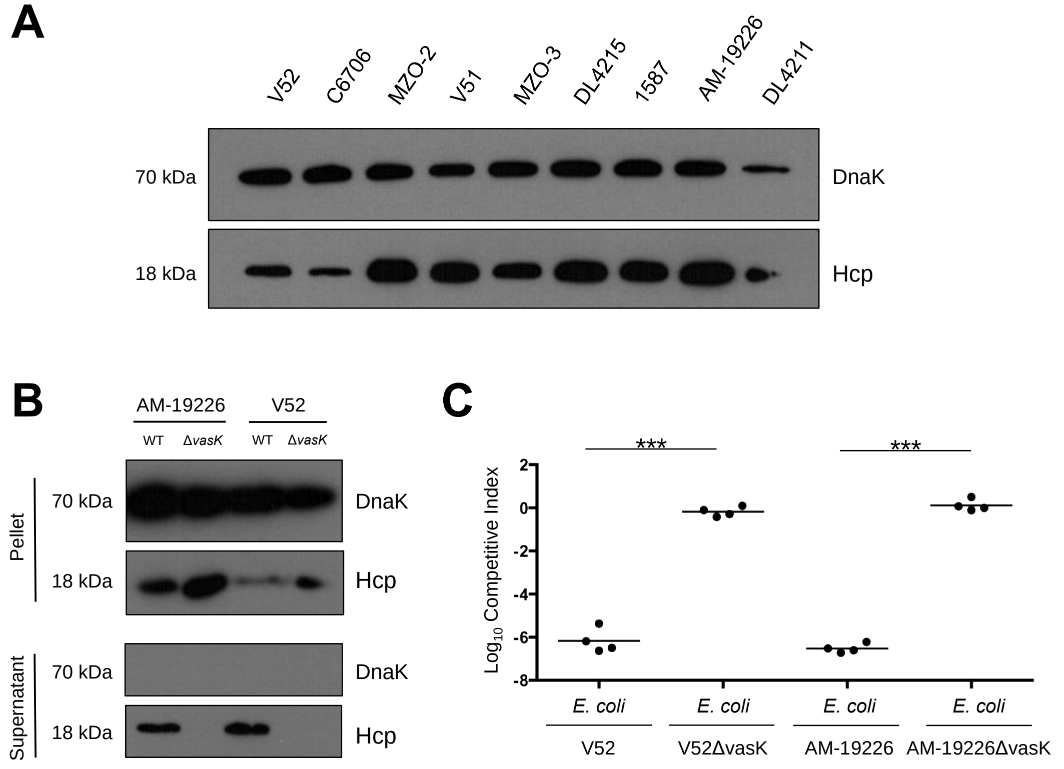

Supplementary Figure 2. *V. cholerae* strains that express Hcp under laboratory conditions. (A) Strains were spotted on an LB plate at a density of 10^8 bacteria/25 μL spot. Levels of Hcp and DnaK in whole cell lysates is shown. (B) Strains were grown in liquid LB broth to mid-logarithmic phase under laboratory conditions. After centrifugation, pellets and supernatants were analysed by western-blotting with polyclonal rabbit anti-Hcp antiserum[1] (diluted 1:500) and monoclonal mouse anti-DnaK antibody (Stressgen, diluted 1:15,000). (C) A competition assay of *V. cholerae* strains V52 and AM-19226 (wild-type and Δ*vasK* mutants) against *E. coli* at a 10:1 ratio. The results of two independent experiments that were each performed in duplicate are shown. Horizontal bars represent the arithmetic mean of log-transformed data. Error bars indicate the standard deviation of log-transformed data. Stars indicate statistical significance (unpaired, two-tailed student's t-test: $t_6=19.75$, $P<0.0001$ and $t_6=38.55$, $P<0.0001$).

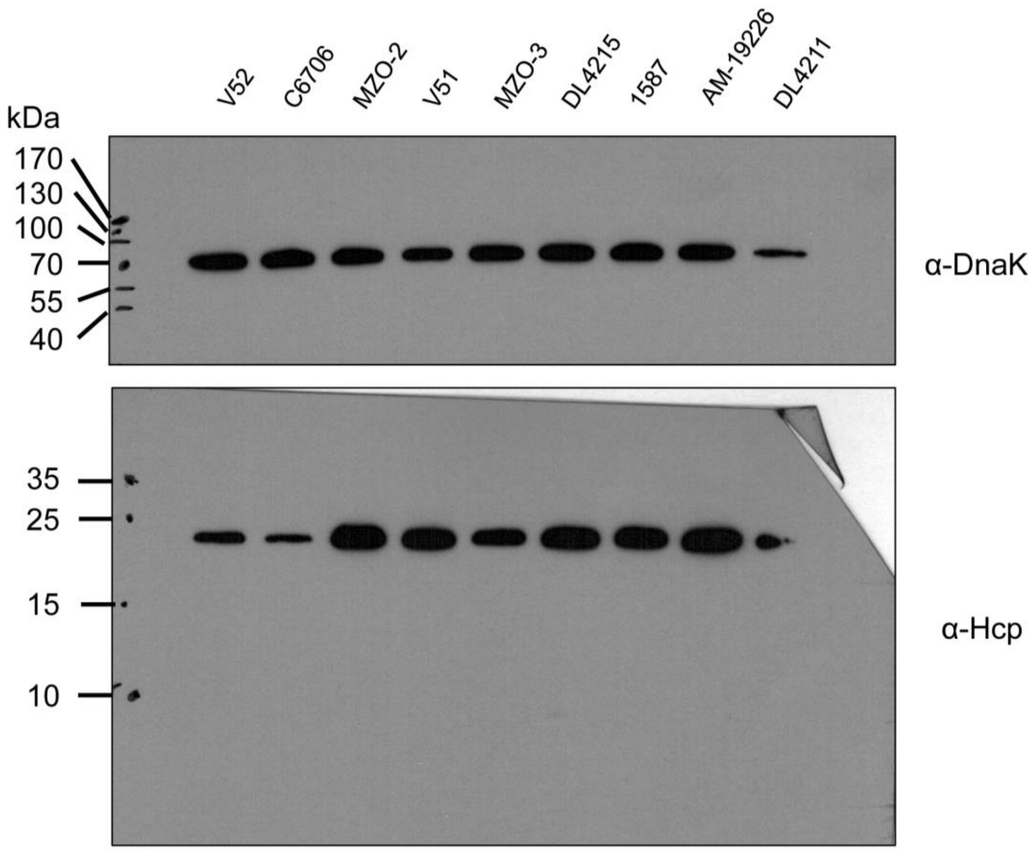

Supplementary Figure 3. Full blot of Supplementary Fig. 2A. Analysis of Hcp expression by SDS page. Expression of DnaK was analyzed as a loading control. Samples were loaded onto one 15% SDS polyacrylamide gel. After transfer to a nitrocellulose membrane, the membrane was cut horizontally between the 35kDa and 40kDa molecular weight mark.

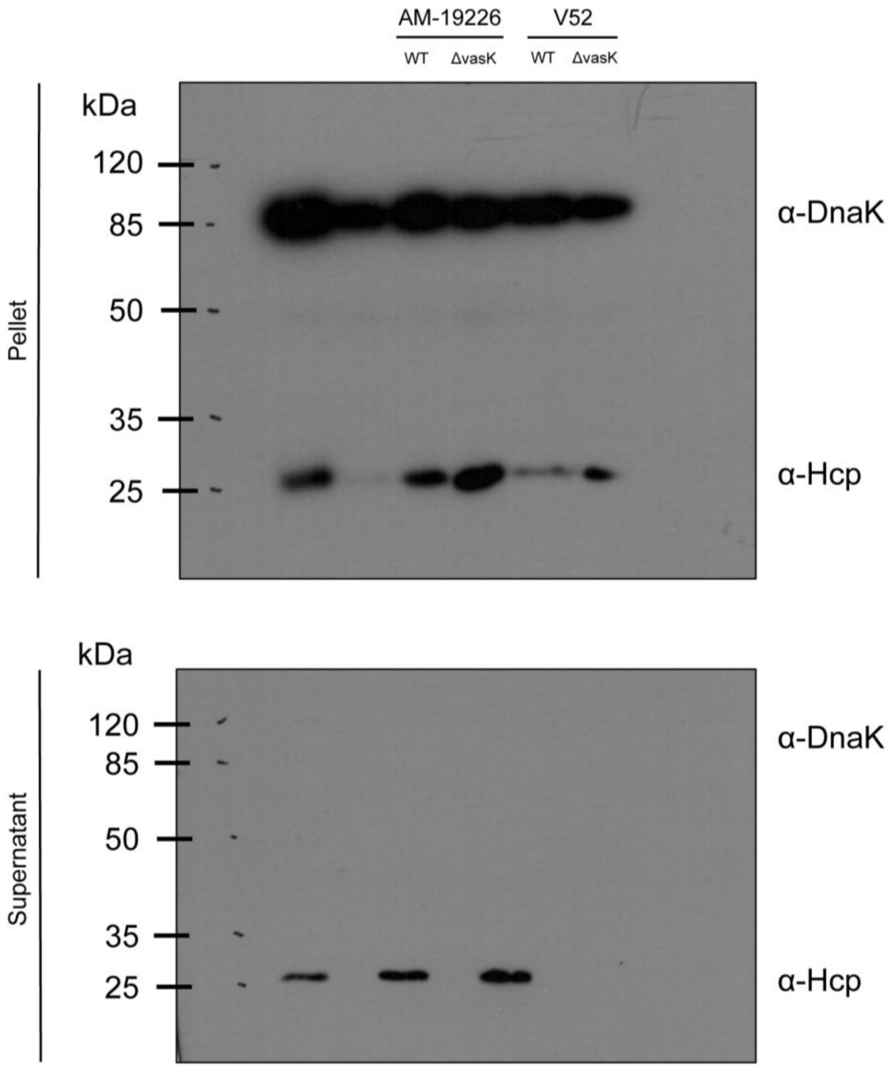

Supplementary Figure 4. Full blot of Supplementary Fig. 2B. Analysis of DnaK and Hcp expression in pellet and supernatant samples by SDS-PAGE.

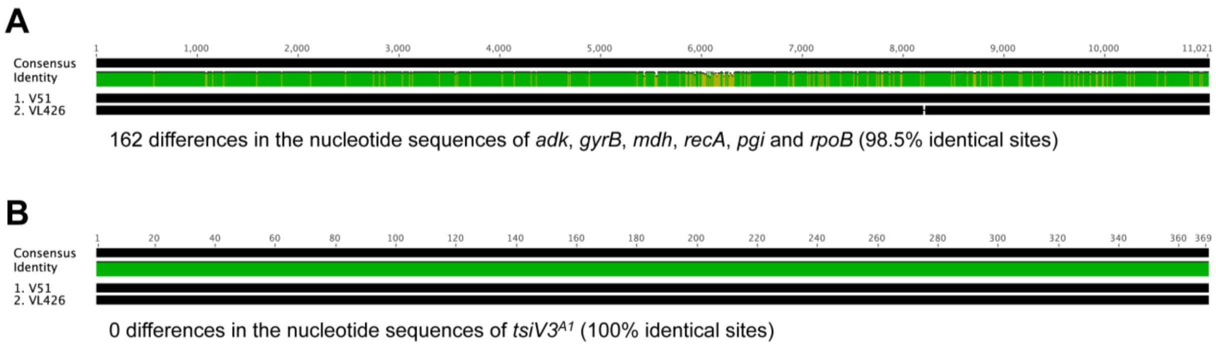

Supplementary Figure 5. Variability in housekeeping genes but not in *tsiV3* between the strains V51 and VL426. (A) Alignment of the concatenated nucleotide sequences of the genes *adk, gyrB, mdh, recA, pgi* and *rpoB.* Green bars correspond to 100%, yellow bars to less than 100% identity between the nucleotides of the two compared sequences. The strain names are indicated on the left. (B) Alignment of the nucleotide sequences of *tsiV3*^{A1} of V51 and VL426.

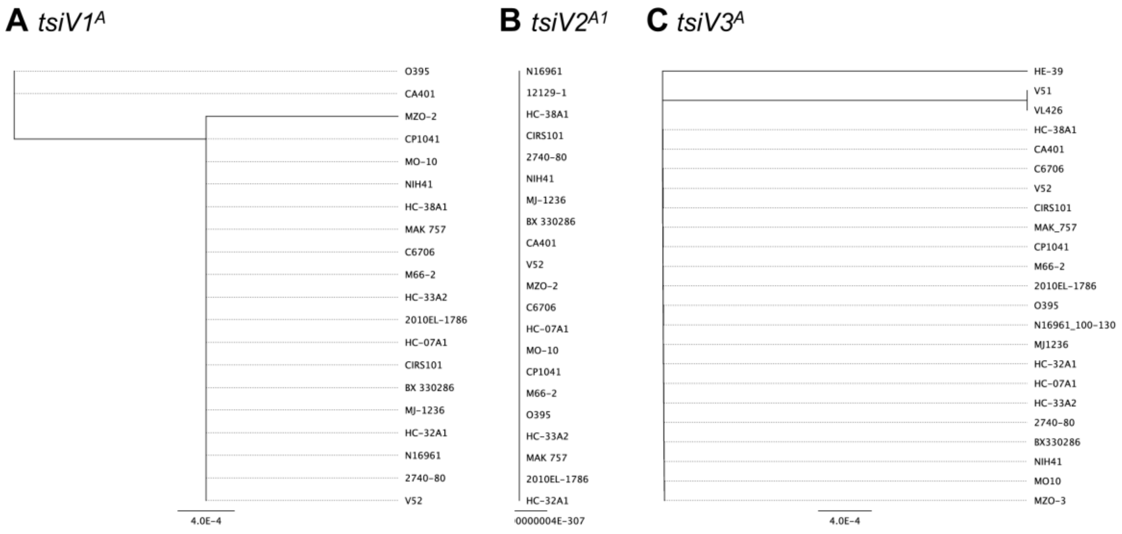

Supplementary Figure 6. Genes *tsiV1*^{A}, *tsiV2*^{A1}, and *tsiV3*^{A} differ in their distribution and phylogeny. Phylogenetic trees of *tsiV1*^{A} (A), *tsiV2*^{A1} (B), and *tsiV3*^{A} (C) of indicated strains.

Supplementary Table 1. *V. cholerae* strains used in this study

Strain	Module Set	TCP/CTX	Serogroup (Biotype)	Isolation	Year	Location
V51	$C_3D_3A_1$	-/+	O141	Clinical	1987	USA
1587	$C_3D_2C_1$	-/-	O12	Clinical	1994	Peru
DL4211	$C_6E_2E_1$	-/-	O123	Environment	2008	Rio Grande/USA
VL426	$C_8A_4A_1$	-/-	nonO1/O139	Environment	?	Germany
MZO-2	$A_3A_1B_1$	-/-	O14	Clinical	2001	Bangladesh
12129(1)	$C_1A_1D_1$	-/-	O1 (ElTor, Inaba)	Environment	1985	Australia
LMA3984-4	$B_1A_3C_1$	-/-	O1 (Ogawa)	Environment	2007	Brazil
623-39	$C_5D_1C_1$	-/-	nonO1/O139	Environment	2002	Bangladesh
AM-19226	$C_1D_4F_1$	-/-	O39	clinical	2001	Bangladesh
HE-25	$D_1D_5F_3$	-/-	?	Env. Gray Water	2010	Haiti
HE-39	$C_7D_1A_2$	-/-	nonO1/O139	Environment	2010	Haiti
HE-45	$C_7C_1D_1$	-/-	?	Env. Gray Water	2010	Haiti
HC-43B1	$C_7A_2D_1$	-/-	O1 (ElTor, Ogawa)	Clinical	2010	Haiti
Amazonia	$C_5C_1G_1$	-/-	O1	Clinical	1991	Brazilian Amazon
TM11079-80	$C_5C_1G_1$	-/-	O1 (ElTor, Ogawa)	Sewage	1980	Brazil

DL4215	$C_4E_1C_2$	-/-	O113	Environment	2008	Rio Grande/USA
TMA21	$C_2B_1F_2$	-/-	nonO1/O139	Environment	1982	Brazil
MZO-3	$C_1E_1A_1$	-/-	O37	Clinical	2001	Bangladesh
2740-80	$A_1A_1A_1$	-/-	O1 (ElTor, Inaba)	Environment	1980	Gulf Coast/ USA
M66-2	$A_1A_1A_1$	+/-	O1	Clinical	1937	Indonesia
BX 330286	$A_1A_1A_1$	+/+	O1 (ElTor, Inaba)	Environment	1986	Australia
MAK757	$A_1A_1A_1$	+/+	O1 (ElTor)	Clinical	1937	Indonesia
CIRS 101	$A_1A_1A_1$	+/+	O1 (ElTor, Inaba)	Clinical	2002	Bangladesh
CP1041	$A_1A_1A_1$	+/+	O1 (ElTor, Ogawa)	Clinical	2004	Zimbabwe
HC-07A1	$A_1A_1A_1$	+/+	O1	Clinical	2010	Haiti
HC-32A1	$A_1A_1A_1$	+/+	O1 (ElTor, Ogawa)	Clinical	2010	Haiti
HC-33A2	$A_1A_1A_1$	+/+	O1 (ElTor, Ogawa)	Clinical	2010	Haiti
HC-38A1	$A_1A_1A_1$	+/+	O1 (ElTor, Ogawa)	Clinical	2010	Haiti
MO10	$A_1A_1A_1$	+/+	O139	Clinical	1992	India
N16961	$A_1A_1A_1$	+/+	O1 (ElTor, Inaba)	Clinical	1975	Bangladesh
2010EL-1786	$A_1A_1A_1$	+/+	O1 (ElTor, Ogawa)	Clinical	2010	Haiti
C6706	$A_1A_1A_1$	+/+	O1 (ElTor,	Clinical	1991	Peru

			Inaba)			
MJ1236	$A_1A_1A_1$	+/+	O1 (ElTor, Inaba)	Clinical	1994	Bangladesh
V52	$A_1A_1A_1$	+/+	O37	Clinical	1968	Sudan
CA401	$A_2A_1A_1$	+/+	O1 (Classical)	Clinical	1953	India
O395	$A_2A_1A_1$	+/+	O1 (Classical)	Clinical	1965	India
NIH41	$A_1A_1A_1$	+/+	O1 (Classical)	?	1940	India

Supplementary Table 2. Reference or source of strains used in this study

Strain	Description	Reference or Source
V. cholerae V52	O37 serogroup strain, Sm^R	Dr. J. Mekalanos, Harvard Medical School[2]
V. cholerae V52	O37 serogroup strain, Rif^R	Dr. J. Mekalanos, Harvard Medical School
V. cholerae V52Δ*vasK*	V52 mutant lacking *vasK* (VCA0120), Sm^R	[3]
V. cholerae V52Δ*vasK*	V52 mutant lacking *vasK* (VCA0120), Rif^R	This study
V. cholerae V52Δ*vasX*Δ*vgrG3*	V52 mutant lacking *vasX* (VCA0020) and *vgrG3* (VCA0123)	[3]
V. cholerae V52Δ*tseL*Δ*vgrG3*	V52 mutant lacking *tseL* (VC1418) and *vgrG3* (VCA0123)	[3]
V. cholerae V52Δ*tseL*Δ*vasX*	V52 mutant lacking *tseL* (VC1418) and *vasX* (VCA0020)	[3]
V. cholerae V52Δ*tseL*Δ*vasX*Δ*vgrG3*	V52 mutant lacking *tseL* (VC1418) and *vasX* (VCA0020) and *vgrG3* (VCA0123)	[3]
V. cholerae C6706	O1 El Tor strain, Sm^R, Rif^R	Dr. J. Mekalanos, Harvard Medical School
V. cholerae C6706Δ*tsiV2*Δ*tsiV3*	C6706 mutant lacking *tsiV2* (VCA0021) and *tsiV3* (VCA0124)	This study
V. cholerae C6706Δ*tsiV1*Δ*tsiV3*	C6706 mutant lacking *tsiV1* (VC1419) and *tsiV3* (VCA0124)	This study

V. cholerae C6706Δ*tsiV1*Δ*tsiV2*	C6706 mutant lacking *tsiV1* (VC1419) and *tsiV2* (VCA0021)	This study
V. cholerae C6706Δ*tsiV1*Δ*tsiV2*Δ*tsiV3*	C6706 mutant lacking *tsiV1* (VC1419) and *tsiV2* (VCA0021) and *tsiV3* (VCA0124)	This study
V. cholerae AM-19226Δ*endonuclease*	Non-O1, non-O139 strain	Dr. J. Mekalanos, Harvard Medical School[4]
V. cholerae AM-19226Δ*endonuclease*Δ*vasK*	AM-19226 mutant lacking *vasK* (VCA0120)	This study
V. cholerae MZO-2	O14 serogroup strain, Sm^R	Dr. J. Zhu, University of Pennsylvania
V. cholerae V51	O141 serogroup strain	Dr. M. Dziejman, University of Rochester
V. cholerae MZO-3	O37 serogroup strain, Sm^R	Dr. M. Dziejman, University of Rochester
V. cholerae DL4215	O113 serogroup strain, Sm^R	Dr. D. Provenzano, University of Brownsville, Texas
V. cholerae 1587	O12 serogroup strain	Dr. M. Dziejman, University of Rochester
V. cholerae DL4211	O123 serogroup strain, Sm^R	Dr. D. Provenzano, University of Brownsville, Texas
V. cholerae N16961	El Tor O1 serogroup strain, Sm^R	Dr. J. Mekalanos, Harvard Medical School
V. cholerae O395	Classical O1 serogroup strain, Sm^R	Dr. J. Mekalanos, Harvard Medical School
V. cholerae MO10	O139 serogroup strain, Sm^R	Dr. M. Dziejman, University of Rochester

V. cholerae MAK757	El Tor O1 serogroup strain	Dr. M. Dziejman, University of Rochester
V. cholerae 2740-80	El Tor O1 serogroup strain, Sm^R	Dr. J. Zhu, University of Pennsylvania
Escherichia coli MG1655	Rif^R	[5]

Supplementary Table 3. Primers used in this study

Name	Direction	Sequence	Function
VCA0120-A	F	ACTAGT CTG TCG ATT CGT CTG CG	Knock-out *vasK* AM-19226
VCA0120-B	R	CCTATTAATAGAGTGTTTTAATGAATTTCCACATGAAT	Knock-out *vasK* AM-19226
VCA0120-C	F	ATTCATGTGGAAATTCATTAAAACACTCTATTAATAGG	Knock-out *vasK* AM-19226
VCA0120-D	R	ACTAGT AGTGCAACCCC	Knock-out *vasK* AM-19226

Primers used to make knock-out constructs for *tsiV1*, *tsiV2*, *tsiV3*, *tseL*, *vasX* and *vgrG3* have been described before[3].

Supplementary Table 4. GenBank accession numbers of strains used in this study

V. cholerae strain	GenBank Accession
12129(1)	ACFQ00000000
1587	AAUR01000000
2740-80	AAUT01000000
623-39	AAWG00000000
2010EL-1786	CP003069/CP003070
AM-19226	AATY01000000
Amazonia	AFSV00000000
BX 330286	ACIA00000000
C6706	AHGQ00000000
CA401*	KF228943/KF228947/KF228950
CIRS101	ACVW00000000
CP1041	SRA037374
DL4211*	KC955251/KF228941/KF228945
DL4215*	KF228942/KF228946/KF228949
HC-07A1	SRA035959
HC-32A1	SRA035998
HC-33A2	SRA035995
HC-38A1	SRA030739
HC-43B1	ALDP01000000
HE-25	ALEC00000000
HE-39	SRA030720
HE-45	ALED00000000
LMA3984-4	CP002555/CP002556
M66-2	NC_012578/NC_012580

MAK 757	AAUS00000000
MJ-1236	CP001485/CP001486
MO10	AAKF03000000
MZO-2	AAWF01000000
MZO-3	AAUU01000000
N16961	AE003852/AE003853
NIH41*	KF228944/KF228948/KF228951
O395	CP000626/CP000627
TM 11079-80	ACHW00000000
TMA21	ACHY00000000
V51	AAKI02000000
V52	AAKJ02000000
VL426	ACHV00000000

(Strains for which sequences of T6SS gene clusters were generated in this study and are now available under the indicated accession codes are marked with an asterisk).

Supplementary Table 5. Additional information for the statistical analysis

Figure	Analysed	Test statistic, degree of freedom in subscript	P-value	
4	Panel 2, bar 2/5	t_6=3.657	P=0.0106	Two-tailed
4	Panel 3, bar 3/5	t_6=12.08	P<0.0001	Two-tailed
4	Panel 4, bar 4/5	t_6=8.584	P=0.0001	Two-tailed
4	Panel 5, bar 1/2	t_6=1.146	P=0.2956	Two-tailed
4	Panel 5, bar 2/5	t_6=0.1280	P=0.9024	Two-tailed
4	Panel 5, bar 3/5	t_6=0.1200	P=0.9084	Two-tailed
4	Panel 5, bar 4/5	t_6=0.5422	P=0.6072	Two-tailed
5A	Column 1	t_6=0.5235	P=0.6194	Two-tailed
5A	Column 2	t_6=2.337	P=0.0581	Two-tailed
5A	Column 3	t_6=16.19	P<0.0001	Two-tailed
5A	Column 4	t_6=12.09	P<0.0001	Two-tailed
5A	Column 5	t_6=29.15	P<0.0001	Two-tailed
5A	Column 6	t_6=10.28	P<0.0001	Two-tailed
5A	Column 7	t_6=30.28	P<0.0001	Two-tailed
5A	Column 8	t_6=7.267	P=0.0003	Two-tailed
5A	Column 9	t_6=28.30	P<0.0001	Two-tailed
5B	Column 1	t_6=0.5968	P=0.5725	Two-tailed
5B	Column 2	t_6=0.01607	P=0.9877	Two-tailed
5B	Column 3	t_6=16.01	P<0.0001	Two-tailed
5B	Column 4	t_6=0.8371	P=0.4346	Two-tailed
5B	Column 5	t_6=2.371	P=0.0555	Two-tailed

5B	Column 6	t_6=0.1582	P=0.8795	Two-tailed
5B	Column 7	t_6=1.358	P=0.2234	Two-tailed
6A	Column 1/4	t_6=15.44	P<0.0001	Two-tailed
6A	Column 2/4	t_6=19.66	P<0.0001	Two-tailed
6A	Column 3/4	t_6=13.18	P<0.0001	Two-tailed

Supplementary References:

1 Ma, A. T., McAuley, S., Pukatzki, S. & Mekalanos, J. J. Translocation of a *Vibrio cholerae* type VI secretion effector requires bacterial endocytosis by host cells. *Cell Host & Microbe* **5**, 234-243, doi:10.1016/j.chom.2009.02.005 (2009).

2 Zinnaka, Y. & Carpenter, C. C., Jr. An enterotoxin produced by noncholera *Vibrios*. *The Johns Hopkins Medical Journal* **131**, 403-411 (1972).

3 Miyata, S. T., Unterweger, D., Rudko, S. P. & Pukatzki, S. Dual expression profile of type VI secretion system immunity genes protects pandemic *Vibrio cholerae*. *PLoS Pathogens* **9**, e1003752, doi:10.1371/journal.ppat.1003752 (2013).

4 Tam, V. C., Serruto, D., Dziejman, M., Brieher, W. & Mekalanos, J. J. A type III secretion system in *Vibrio cholerae* translocates a formin/spire hybrid-like actin nucleator to promote intestinal colonization. *Cell Host & Microbe* **1**, 95-107, doi:10.1016/j.chom.2007.03.005 (2007).

5 MacIntyre, D. L., Miyata, S. T., Kitaoka, M. & Pukatzki, S. The *Vibrio cholerae* type VI secretion system displays antimicrobial properties. *Proceedings of the National Academy of Sciences of the United States of America* **107**, 19520-19524, doi:10.1073/pnas.1012931107 (2010).
